# The exercise paradox: An interactional model for a clearer conceptualization of exercise addiction

**DOI:** 10.1556/JBA.2.2013.4.2

**Published:** 2013-12-13

**Authors:** Alexei Y Egorov, Attila Szabo

**Affiliations:** ^1^Department of Psychiatry and Addictions, Faculty of Medicine, St. Petersburg State University, St. Petersburg, Russia; ^2^Department of Psychiatry and Addictions, Mechnikov North-West Medical University, St. Petersburg, Russia; ^3^Laboratory of Behaviour Neurophysiology, and Pathology, Sechenov Institute of Evolutionary Physiology and Biochemistry, St. Petersburg, Russia; ^4^Institute for Health Promotion and Sport Sciences, Eötvös Loránd University, Budapest, Hungary; ^5^Institutional Group on Addiction Research, Institute for Psychology, Eötvös Loránd University, Budapest, Hungary

**Keywords:** dependence, exercise abuse, research, review, theory, transformation

## Abstract

*Background and aims:* Exercise addiction receives substantial attention in the field of behavioral addictions. It is a unique form of addiction because in contrast to other addictive disorders it is carried out with major physical-effort and high energy expenditure. *Methods:* A critical literature review was performed. *Results:* The literature evaluation shows that most published accounts report the levels of *risk* for exercise addiction rather than actual cases or *morbidities*. The inconsistent prevalence of exercise addiction, ranging from 0.3% to 77.0%, reported in the literature may be ascribed to incomplete conceptual models for the morbidity. Current explanations of exercise addiction may suggest that the disorder is progressive from healthy to unhealthy exercise pattern. This approach drives research into the wrong direction. *Discussion:* An interactional model is offered accounting for the adoption, maintenance, and transformation of exercise behavior. The here proposed model has an idiosyncratic *black-box* containing the antecedents and characteristics that are unique to the individual, which cannot be researched via the nomothetic approach. Subjective aspects in the black-box interact with stressful life events that force the person to cope. The range of coping may be wide. Escape into exercise depends on personal (subjective) and situational (objective) factors, but the subjective components are inaccessible for *a priori* scholastic scrutiny. It is our view that currently only this dual interactional model may account for the fact that exercise addiction emerges suddenly and only in a few individuals from among those at high risk, estimated to be around 3.0% of the exercising population.

## INTRODUCTION

### Exercise addiction within behavioral addictions

Currently three types of addictions are known in the scholastic literature: 1) substance or chemical addictions, 2) behavioral (non-chemical or non-pharmacological) addictions, and 3) food addictions (Egorov, 2013). Behavioral addictions are compulsive psychological and physiological urges for one or more specific behaviors. There is increased attention devoted to this group of morbidities by researchers in the field. A category of “Behavioral Addiction” is included into the new DSM-V (American Psychiatric Association, 2013). While there are many forms of behavioral addictions with wide range of consequences, the DSM-V category of behavioral addictions only includes gambling addiction. Internet addiction was also considered for inclusion into this category, but there was no consensus in the working group. Therefore, Internet addiction is only included in the manual's appendix to encourage further research. Exercise addiction, after over four decades of scholastic interest and research on the topic, is still left out from the DSM-V.

It is becoming increasingly more evident that people could become addicted to various behaviors. In addition to behavioral addiction to gambling recognized in the DSM-V (Parke & Griffiths, 2004), new forms of addictive behaviors have surfaced in the medical literature. Indeed, numerous individuals spend too much time at work (workaholism – Scottl, Moore & Miceli, 1997), or online (Internet addiction – Young, 1998). Some are too fond of shopping (shopping addiction – Krueger, 1988), watching television (addiction to television – Kubey & Csikszentmihalyi, 2002), overindulging in porn or sexual activity (sexual addiction – Carnes, 1983), or physical activity (exercise addiction – Szabo, 2010), or even tango dancing (dance addiction – Targhetta, Nalpas & Perney, 2013). While to date relatively little attention has been devoted to the understanding and treatment of these “imprisoning” behaviors, they should no longer be ignored. As pointed out by Martin and Petry (2005), non-chemical addictions may not only resemble, but they also share a common neurobiological mechanism with alcohol or drug addictions. Thus behavioral addictions are not only possible, but they are preponderant in the daily human life (Holden, 2001) – regardless of the recognition and/or consensus of the DSM-V working group – and may be more common than expected, because it is difficult to detect them as they very often blend into the *normal* spectrum of the daily human activities. Indeed, behavioral addictions are often as serious in their consequences as alcohol or drug addictions (Martin & Petry, 2005). A majority of non-pharmacological addictions are usually encountered within a family context and often seem to be fostered by family processes (J.Y. Yen, C.F. Yen, C.C. Chen, S.H. Chen & Ko, 2007). Therefore, family therapy is usually a first option in treating the variety of non-pharmacological addictions at individual-clinical (idiosyncratic cases), rather than group-research level.

**Table 1.** Working classification of non-chemical forms of addictive behavior (Egorov, 2007, 2013)

Gambling addiction	Erotic addiction	Positive (or socially accepted) addictions (Glasser, 1976)	Technological addictions	Food addictions
Gambling and betting addictions	Love addiction	Workaholism or work addiction	Internet addiction^1^	Overeating addiction
	Sexual addiction	Exercise addiction^2^	Mobile phone addiction	Starvation-diet related addiction
	Mixed love-sex, partner addiction	Shopping addiction (compulsive buying)	Television addiction	
	Pornography addiction	Religious addiction		
		Relationship addiction		

1 Internet addiction includes: Internet-gamblers, Internet-gamers, Internet-workaholics, Internet-sexaholics, Internet-erotaholics, Internet-shopaholics, Internet relationship and social networking addicts.

2 Exercise addiction does not include excessive exercise observed as symptom in eating disorders.

There are several new attempts to classify behavioral addictions. Egorov (2007, 2013) offered a working classification of non-chemical forms of addictive behavior (Table 1). In this classification Glasser's (1976, 2012) concept of positive addiction has been incorporated in a context of mundane humane behaviors that under ordinary circumstances may make the individual stronger and happier. These behaviors, however, turn into *negative addictions* or psychopathology once they start to be abused to the point where they result in harm to both the affected individual and her/his social surroundings.

Physical exercise is one of the behaviors that benefits people both physically and mentally and, therefore, its regular practice may be beneficial and viewed by Glasser (2012) – and perhaps many others – as therapeutic. Recently, Glasser highlighted that in certain cases self-improving behaviors like exercise or meditation could become addictive and this form of addiction builds strength in the person and promotes a happier and healthier living. This is a view that in conjunction with the clinically diagnosed cases of exercise addiction (Griffiths, 1997) confers a paradoxical role for exercise behavior. Indeed, habitual or committed forms of exercise may be therapeutic, while loss of control renders the behavior pathogenic. In this analytical account the current models forwarded for exercise addiction are reconsidered. Then in an attempt to segregate *risk assessment* (nomothetic approach via questionnaire-screening) and clinical *diagnosis* of exercise addiction, an alternative interactional model is proposed for the better understanding of the exercise paradox.

## EXERCISE ADDICTION

In the past decades several publications dealing with exercise addiction have emerged. Research into highly accustomed exercise started with a work that investigated the effects of exercise deprivation on sleep patterns (Baekeland, 1970). The author of the study faced great difficulties in recruiting highly committed athletes (exercising 5–6 days a week), who would be willing to give up their training for one month. In fact, eligible potential participants have refused to participate in the experiment even when they were offered cash reward. Baekeland was only able to recruit athletes who trained only 3–4 times a week. During the month of the deprivation, these participants reported negative psychological well-being, which surfaced as high level of anxiety, frequent night awakenings, and sexual tension.

Later the concept of addiction to exercise was first introduced by Sachs and Pargman (1984). The authors have used the term *running addiction* to describe the source of a set of withdrawal symptoms that surface during periods of running deprivation: anxiety, tension, irritability, muscle twitching, etc. However, earlier, Morgan (1979) also provided examples in which runners continued to run, despite the adverse circumstances (for example, various injuries), which should reduce or interrupt training. Diagnosed clinical cases of exercise addiction in all kinds of sports – martial arts, weight lifting, and body building – were only reported later (Griffiths, 1997; Hurst, Hale, Smith & Collins, 2000; Murphy, 1994). These clinical cases of exercise addiction are characterized by loss of control over the exercise behavior, which is performed as “obligation” rather than for enjoyment, and also *have negative physical and psychosocial consequences* for the individual. Symptoms include all components of addictive disorders: salience, withdrawal, mood modification, conflict, tolerance, and relapse (Szabo, 2010). In light of this definition pathogenic exercisers could be distinguished from the other high-volume exercisers, like athletes, who maintain control over their training, have a fixed schedule of training to also meet other life-obligations, and encounter no harmful or negative consequences as a result of their intensive training.

To avoid a conceptual confound, it should be mentioned that De Coverley Veale (1987) differentiated between primary and secondary exercise addiction. In this article only primary exercise addiction is considered because secondary exercise addiction is a symptom in a number of eating disorders including Anorexia Nervosa and Bulimia Nervosa (De Coverley Veale, 1987). In these disorders, excessive exercise is a means for caloric control and weight loss rather than for escape from a psychological hardship. Secondary exercise addiction as a symptom in eating disorders occurs in different “doses” in people affected by eating disorders. It was estimated that one third of anorectics may be affected (Crisp, Hsu, Harding & Hartshorn, 1980).

## ESTIMATED PREVALENCE OF EXERCISE ADDICTION

Mass screening for exercise addiction takes place by using psychometrically validated questionnaires. Two instruments that prove to be similar in sensitivity and reliability (Mónok et al., 2012) are the Exercise Dependence Scale (EDS, 21 items; Hausenblas & Downs, 2002) and the Exercise Addiction Inventory (EAI, 6 items; Terry, Szabo & Griffiths, 2004). These scales do not convey exact – or accurate – information about the *actual* prevalence of exercise addictions since they are screening- rather than diagnosis-tools. Indeed, the estimates based on these questionnaires should be interpreted as symptomatic or *at risk for exercise addiction* as also noted be the developers of the tools (Hausenblas & Downs, 2002; Terry et al., 2004).

A number of inquiries were conducted on convenience samples of university students. Hausenblas and Downs (2002) reported that between 3.4% and 13.4% of their samples were at high risk for exercise addiction. The lower figure was also confirmed by Griffiths, Szabo and Terry (2005) who reported that 3.0% of university students could be at-risk of exercise addiction. Later Szabo and Griffiths (2007) confirmed that the prevalence of risk for exercise addiction is about 3.6% in the general exercising population, while the figure is nearly double (6.9%) in British Sport Science undergraduates. The study by Hausenblas and Downs (2002) was conducting by using the EDS, whereas the other two by using the EAI. Nevertheless, the two instruments yielded comparable results in American and British samples. Recently, in a Hungarian population-wide study (Mónok et al., 2012) the proportion of exercisers at-risk for addiction was 1.9% among exercisers and 0.3% in the general population as gauged with the EDS. However, the EAI yielded slightly higher figures, 3.2% in habitual exercisers and 0.5% in the general population. Mónok et al. (2012) attributed the discrepancy to a lack of an empirically established cut-off point for the EAI. In spite of the slight discrepancy between the EDS and EAI they appear to project a “close estimation” of the prevalence of risk for exercise addiction in committed exercisers (Sussman, Lisha & Griffiths, 2011a). Several investigations that used other instruments than the EDS and EAI in the scrutiny of the risk for exercise addiction have found exaggerated or unlikely figures for the morbidity, as summarized in Table 2.

Although researchers have stressed that actual cases of exercise addiction are rare (Szabo, 2000; Veale, 1995) especially when compared to other addictions (Sussman et al., 2011b), figures of above 40% prevalence, published in the past five years, suggest that the psychopathology is not well understood among scholars. The diversity in instruments used, samples, and methods of inquiry – as well as some possible cross-cultural issues that were not addressed to date – may all contribute to the inconsistencies seen in Table 2. Further, as noted earlier, the questionnaire-based studies could only estimate the preponderance of the “at-risk” exercisers rather than actual clinical cases. Consequently, the latter may be even lower than the estimates based on the population-wide results reported recently (Mónok et al., 2012).

## THEORETICAL MODELS FOR EXERCISE ADDICTION

**Figure 1.**
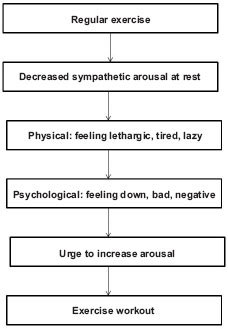
The Sympathetic Arousal Hypothesis

The apparent lack of understanding of the exercise paradox, begs for sound theory-driven research. In his 2010 monograph, Szabo presented two specific models for exercise addiction and several models that try to explain the psychological beneficence of exercise, which in turn could be indirectly linked to exercise addiction. In the current paper only the specific models are dealt with. The Sympathetic Arousal Hypothesis (Thompson & Blanton, 1987) is physiological model suggesting how adaptation of the organisms to habitual exercise may lead to addiction. Briefly, adaptation to exercise lowers the body's sympathetic activity. Lower sympathetic activity at rest means lower level of arousal. This new baseline or resting level of arousal may not be adequate for various daily activities. It may be experienced as a lethargic or energy-lacking state. This feeling prompts the person to do something about it, or to increase her/his arousal. One means to do that is exercise. However, the effects of exercise in increasing arousal are only temporary and, therefore, more and more bouts of exercise may be needed to trigger an optimal state of arousal (Figure 1). Further, not only the frequency but also the volume of exercise may need to be increased due to training effect. Such an increase accounts for the tolerance in the addiction process. The main dilemma with this model is that sympathetic adaptation to exercise is universal, so it occurs in everyone, but only about 3% of the regular exercises may become addicted to the behavior (Sussman et al., 2011a).

**Figure 2.**
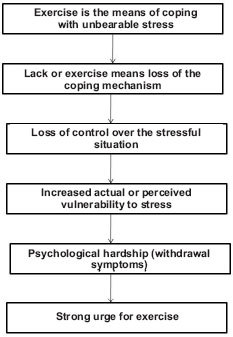
The Cognitive Appraisal Hypothesis

The second model presented by Szabo (2010) was the Cognitive Appraisal Hypothesis (Szabo, 1995). This model takes in consideration life-stress – that requires challenge beyond one's perceived resources – in the addiction model. Some (but it is unknown who) exercisers may try to escape from an ongoing or a sudden stress by resorting to exercise as the means of coping with stress. Once exercise is the coping method with stress, the person depends on it to function well. She/he believes that exercise is a healthy means of coping with stress based on information from scholastic and public information sources. Therefore, the person is using rationalization to explain the exaggerated amount of exercise that progressively takes a tool on other obligations and daily activities. However, when interference of exercise with other life-obligations forces the individual to reduce the amount of exercise, psychological hardship emerges in form of withdrawal symptoms. Loss of exercise also means the loss of the coping mechanism. Consequently, the exerciser loses control, which generates greater vulnerability to stress by further amplifying the negative feelings associated with the lack of exercise. The problem could be resolved only through resuming the previous pattern of exercise often at the expense of the other obligations in the daily life (Figure 2). While this model depicts exercise addiction as coping or escape, it only accounts for *maintenance* of addiction, but not its onset.

*Based on:* Thompson & Blanton (1987).

**Table 2.** Prevalence of exercise addiction according to extant reports in the scholastic literature

Year	Author(s)	Sample studied	Measure(s) used	Prevalence (%)
1995	Thornton & Scott	Runners	Commitment to Running Scale (CRS – Carmack & Martens, 1979)	77%
1998	Slay et al.	Runners	Obligatory Running Questionnaire (Blumenthal, O'Toole & Chang, 1985)	26.2% of male runners, 25% of female runners
2000	Bamber, Cockerill & Carroll	Mixed exercisers and university students	Exercise Dependence Questionnaire (EDQ – Ogden, Veale & Summers, 1997)	14.8% and 9% also suffering of eating disorders
2002	Ackard, Brehm & Steffen	Female university exercisers	Obligatory Exercise Questionnaire (Pasman & Thompson, 1988)	8.0%
2002	Blaydon & Lindner	Triathletes	EDQ	30.4% primary and 20.6% secondary exercise addiction
2002	Hausenblas & Downs	University students	Exercise Dependence Scale (EDS – Hausenblas & Downs, 2002)	3.4% and 13.4% in two studies
2004	Downs, Hausenblas & Nigg	University students	EDS-Revised (EDS-R – Downs et al., 2004)	3.6% and 5.0% in two studies
2005	Griffiths et al.	University students	Exercise Addiction Inventory (EAI – Terry et al., 2004)	3.0%
2007	Allegre, Therme & Griffiths	Ultra-marathoners	EDS-R (French)	3.2%
2007	Szabo & Griffiths	Habitual exercisers and Sport Science students	Exercise Addiction Inventory (EAI – Terry et al., 2004)	3.6% in habitual exercisers, 6.9% in Sport Science undergraduates
2007	Youngman	Triathletes	EAI	19.9%
2008	Lejoyeux, Avril, Richoux,Embouazza & Nivoli	Fitness centre attendees	Interview and own questionnaire	42%
2009	Modolo et al. (cf. Modolo et al., 2011)	Various amateur athletes	Negative addiction Scale (NAS – Hailey & Bailey, 1982)	32%
2010	Smith, Wright & Winrow	Competitive runners	EDS and Running Addiction Scale (RAS – Chapman & De Castro, 1990)	50%
2011	Grandi, Clementi, Guidi, Benassi & Tossani 2011	Habitual exercisers	EDQ (Italian)	40.5%
2011	Villella et al.	High school students	EAI (Italian)	8.5%
2012	Costa, Cuzzocrea, Hausenblas, Larcan & Oliva	Fitness centre attendees	EDS-R (Italian)	6.6%
2012	Lejoyeux, Guillot, Chalvin, Petit & Lequen	Fitness centre attendees	EAI (French) and own questionnaire	29.6%
2012	McNamara & McCabe	Elite athletes	The Exercise Dependence and Elite Athletes Scale (EDEAS – McNamara & McCabe, 2013)	34.8%
2012	Mónok et al.	Population-wide study	EDS and EAI (Hungarian)	0.3% on EDS and 0.5% on EAI in general population; 1.9% on EDS and 3.2% on EAI in regular exercisers
2013	Lichtenstein, Christiansen, Bilenberg & Støving	Exercisers and soccer players	EAI (Danish)	5.8%
2013	Menczel et al.	Fitness centre attendees	EDS & EAI (Hungarian)	1.8% + 1.8% who exhibited both exercise addiction and eating disorders

**Figure 3.**
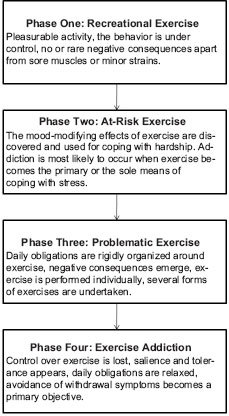
The “Four Phase” model for exercise addiction

A “Four Phase” model for exercise addiction was proposed by Freimuth, Moniz and Kim (2011). The first phase is characterized by pleasurable activity while the behavior is under control. There are no major negative consequences in general, but muscles soreness or minor strains may occur. In phase two, the psychological beneficence of exercise is realized and the mood-modifying effects may be adopted for coping with hardship. Addiction is most likely to occur when exercise becomes the primary or the sole means of coping with stress. This part of the model may address the onset of exercise addiction, but it does not specify two key issues: 1) a distress must exist, whether progressively mounting or suddenly appearing, and 2) under what conditions or influences will exercise be adopted for coping with stress? The third phase is characterized by the rigid organization of daily obligations around exercise, negative consequences due to exaggerated exercise, and several forms of exercise either for replacing or complementing the habitual mode of exercise. Further, exercise is performed individually, rather than with friends, in a team, or during scheduled fitness classes. The fourth or the last stage encompasses the typical symptoms of fully manifested addiction like salience, tolerance, conflict, need for mood modification and the avoidance of withdrawal symptoms and relapse. While the model is appealing indeed, it does not account for the choice of coping mechanism, or who and why from among the exercise population will turn to this form of time and major energy requiring means of coping with adversity (Figure 3).

*Based on:* Szabo (1995).

*Based on:* Freimuth, Moniz & Kim (2011).

**Figure 4.**
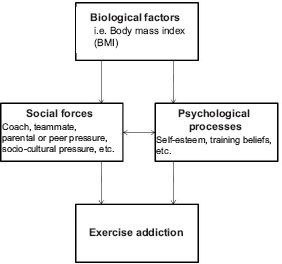
The “Biopsychosocial” model for exercise addiction in elite athletes

A “Biopsychosocial” model for exercise addiction in elite athletes was also proposed recently (McNamara & McCabe, 2012). It is our view that overtraining and over commitment in elite athletes does not parallel the psychiatric cases of exercise addiction. In our opinion this model is questionable for at least two reasons: 1) Timing and availability; If behavioral addictions are means of escape from unbearable stress (Korolenko, 1991), the escape needs to happen when the pain dictates or the urge arises. Elite athletes have a training regimen that is *scheduled for them,* in *group settings,* and at *directed intensity*. These are not characteristics of exercise or any other behavioral or chemical addiction, because the compulsive urges that dominate the person's behavior – after she/he has lost control over the addictive behavior(s) – trigger craving for instant fulfillment. 2) The model has a biologically determined onset, like body mass index (BMI), given as example by the authors (McNamara & McCabe, 2012). If we consider deeply that most addictions are forms of escape from painful reality (Korolenko, 1991), then while biological factors affect psychology, the route of addiction(s) may be – most likely – of psychological origin. The “Biopsychosocial” model (Figure 4) states that exercise addiction has a biological factor (e.g. BMI) at its route of origin in the elite athletes. Social and psychological processes may interact to determine whether exercise addiction will occur or not. Freimuth et al. (2011) warned that intensive training, for long hours, and ambitious strivings to become the best of the best that characterizes successful elite athletes, should not be confused with symptoms of addiction in spite of the fact that there is overlap in the latter. This point of Freimuth et al. is fully endorsed for the two principal reasons discussed above.

*Based on:* McNamara & McCabe (2012).

**Figure 5.**
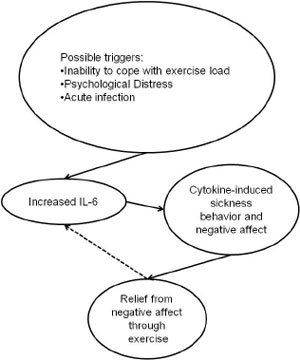
The IL-6 Model for exercise addiction

A theoretical model accentuating the possible role of interleukin six (IL-6) in exercise addiction has been proposed by Hamer and Karageorghis (2007). According to the model, an unidentified trigger causes IL-6 levels to rise and generate cytokine-induced sickness behavior that is linked to negative affect. In individuals affected by psychological hardship an elevated level of IL-6 could yield even more negative mental state. However, the IL-6 hypothesis may not account for the possibility that some individuals will resort to exercise while others may reach for chemical means of escape. The low prevalence of exercise addiction is ascribed to possible adaptations to exercise, whereas the lack of it may increase vulnerability to exercise addiction (Figure 5).

*Based on:* Hamer & Karageorghis (2007).

*Note:* The dotted line reflects that inability to cope with exercise load my further increase IL-6 levels.

The review of the extant models that were specifically forwarded for the explanation of exercise addiction clearly reveals that there is inconsistency in the research perspectives from which this behavioral addiction is examined. Simply and perhaps crudely summarized, according to the Sympathetic Arousal Hypothesis most habitual exercisers may be affected by exercise addiction, a fact that is unlikely with a mean estimate of 3% (Sussman et al., 2011a); the Cognitive Appraisal Hypothesis accounts for exercise addiction only *after* the behavior has been adopted for coping with adversity, and cannot explain who/why chooses exercise as a means of coping; the “Four Phase” model is a hierarchical/developmental model, but again it does not address *when and who* would rely on the mood-moderating effects of exercise for coping and with specific (?) adversities or stress. The way the model may be interpreted is that all exercisers who discover the mood improving and other positive psychological results of exercise may become addicted while coping with stress; The “Biopsychosocial” model that was developed for elite athletes has unconvincing theoretical background in context of the freedom of choice to satisfy craving and urges inherent in addictions. Finally the IL-6 model may be an intermediary in the etiology of exercise addiction, but it cannot account neither for the trigger in raising IL-6 levels nor in exercise-related consequences, since according to the model some exercisers may be affected while others (with adaptation) may not. Therefore, a model accounting for the *adaption, maintenance, and transformation* of the behavior – and therefore addressing the exercise paradox – is needed for a consistent conceptualization and research framework in the understanding of exercise addiction as a clinical morbidity.

## AN EXPANDED INTERACTIONAL MODEL FOR EXERCISE ADDICTION

A missing aspect of the existing models for exercise addiction is the determinant(s) of the choice of exercise as a means of escape from hardship. Here it is strongly stressed, that an interaction between personal values, social image, past exercise experience, and life situation jointly determine whether one will use exercise for coping or resort to other means of dealing with stress. The possible number of interactions between personal and situational factors is so large the each case is idiographic in a mindset resembling a secret “black-box”. The box could only be opened *after diagnosis* with the help of mental health professionals. Indeed, exercise addiction, unlike other chemical and/or behavioral addictions, has a unique characteristic not present in other addictions, which is the physical challenge or work. It was proposed, based on preliminary laboratory evidence, that exercise acts as cathartic-buffer for stress (Tsang & Szabo, 2003). Habitual exercisers when experiencing stress – knowing the mood improving effects of exercise from past experience (Freimuth et al., 2011) – may resort to exercise to cope with the challenge. However, not all exercisers will try to reduce the pain of a novel emotional hardship with exercise, but instead may resort to passive forms of escape behaviors or addiction(s). Therefore, a model taking into account the personal aspects interacting with social-environmental factors may be necessary for the better understanding of the genesis of exercise addiction in the affected individuals. Indeed, a positive relationship was established between exercise addiction *risk-scores* and trait anxiety (Coen & Ogles, 1993), perfectionism (Cook, 1996), and obsessive compulsiveness (Spano, 2001). Further, it was reported that neuroticism, extraversion, and agreeableness could predict symptoms of exercise addiction (Hausenblas & Giacobbi, 2004). Finally, gender (Cook, Hausenblas & Rossi, 2013) and sex role orientation (Rejeski, Best, Griffith & Kenney, 1987) may also have mediating roles. The large combination of subjective psychological factors interacting with situational variables may renders difficult if not impossible the scrutiny of exercise addiction from a nomothetic perspective.

**Figure 6.**
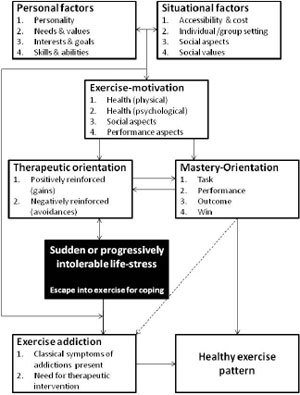
An interactional model for the better understanding of the exercise paradox

The model presented in Figure 6 is an interactional model for exercise addiction. It is in line with the proposed PACE (Pragmatics, Attraction, Communication, Expectation) model for addictions in general (Sussman et al., 2011b). In the current model (Figure 6) a complex set of personal factors interact with a number of environmental – and/or situational – factors to determine the primary motive for exercise behavior. These motives diverge in two directions (Robbins & Joseph, 1985). A health (mental or physical) motivated individual, for example, may run for better or improved health (gain health) and/or to prevent ill health consequences like gaining weight, being lethargic, etc. Both incentives are therapeutic in nature. However, health motives could also have a mastery-orientation, like becoming stronger and lifting more weight (performance orientations), or concentrating better and being more productive at work. If better concentration would be the aim, a therapeutic-orientation would be established, but if the expected *consequence* of the better concentration (productivity) is the objective, then the mastery orientation is the driving force.

**Figure 7.**
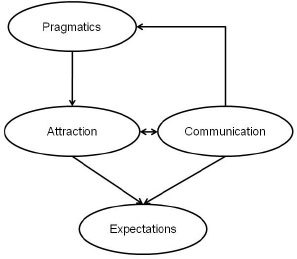
The PACE Model for addictions

The most important component of the here proposed model is the consideration of a suddenly emerging reaction, determined by a set of idiographic (i.e., personal and situational) interactions in the black-box to an ongoing and no longer bearable – or suddenly appearing – life stressor that causes psychological pain over which the individual has no control. This component accounts for the surmise that exercise addiction is not *evolutionary,* or slowly progressing, but rather *revolutionary,* or suddenly surfacing (Szabo, 2010). At the moment when the situation gets out of control, a person will “gravitate” towards a means of available coping in accord with the “Pragmatics” phase of the PACE model (Sussman et al., 2011b – see Figure 7). The choice is determined by conscious and subconscious interactions (in the black-box) between individual aspects, situational factors, and antecedents of exercise behavior, in accord with the “Attraction” component of the PACE model, in a similar way as the motivation for exercise is initially determined. Accordingly, even mastery-oriented exercisers may now shift focus to the therapeutic aspects of exercise and get more involved in it to get rid of the painful stress. This attentional cognition is also in line with the “Communication” factor in the PACE model in that experience, inter- and intrapersonal thought, beliefs and convictions will influence the escape path or the choice of the individual. For example, the lack of experience with alcohol, tobacco, or leisure drugs in conjunction with long exercise history and positive beliefs about exercise (media, social, health values) all interact with unique personal factors during the effort of coping. An already “therapeutic” exerciser in the model is more likely to chose exercise for coping. Then, also in agreement with the PACE model, the greater the expectation from exercise, the more unlikely that the exerciser will turn to other forms of addictions. Being a “positive addiction” it is much easier to hide behind exercise whilst maintaining one's reputation in the social environment, in contrast to other forms of addictions bearing a social stigma.

*Based on:* Sussman et al. (2011b).

The PACE model was proposed for behavioral addictions in general (Sussman et al., 2011b). While the current model is in harmony with the PACE model, it is *specific to exercise* and highlights how orientation, experience and personal-situational interactions could all play mediating roles in the manifestation of exercise addiction. Past research long ago has revealed that addiction risk is higher in those who exercise for escaping the stress or changing their emotions, or physical appearance to improve self-esteem as compared to those who exercise for mastery reasons (Thornton & Scott, 1995). Indeed, Baker, Piper, McCarthy, Majeskie and Fiore (2004) proposed a model for drug addiction in which addictive behavior is sustained through negative reinforcement in an effort to avoid negative affect. Szabo (2010) also argued that exercise addiction is motivated by negative reinforcement. However, initial therapeutic orientation, like losing weight and/or gaining muscles, may – following fulfillment of the goal – turn into mastery orientation and be maintained within the spectrum of healthy exercise pattern. Then, as the bi-directional arrow (refer to Figure 6) indicates between the therapeutic-orientation and major life-stress (black), it is possible that through therapeutic exercising – without addiction – one could master the situation and re-establish a healthy pattern of exercise whilst coping with adversity in a healthy way.

A broken arrow (Figure 6) from mastery orientation to exercise addiction accounts for the unlikely and possibly rare occurrences when an athlete would jeopardize her/his health to stretch the personal limits. It must be stressed that the key reason beyond overtraining – which eventually will be unsuccessful due to strain, injury and staleness – could be traced to mental or psychological origins: 1) the athlete is unable to accept and to realize rationally a personal limit; 2) the athlete strives to beat own or past (other's) record at any cost or otherwise all the athletic career was meaningless, 3) pressure from a past failure, or an unpleasant experience, generates a psychological need to “prove” oneself at whatever cost. While these motivations could fuel exaggerated exercise behaviors, the route and path leading to the manifestations of the behavior is different from that of exercise addiction. In fact, the broken arrow may reflect instances of exaggerated training whilst chasing of dream (or illusion) that an athlete cannot give up. As such, it may be more closely defined as obsessive-compulsive behavior rather than addiction. It should be noted that addiction involves compulsion and dependence (Berczik et al., 2012) and the later may be absent in mastery situations, and therefore marked with a broken arrow.

## CONCLUSION

Approaching half-century of research, exercise addiction is still not well understood. The spectrum of the reported preponderance ranging from 0.3% to 77% shows that there are theoretical and methodological barriers to research in this area. Indeed, nomothetic research could yield results about proneness or risk while actual clinical cases can only be examined through idiographic research. The existing models for exercise addiction are incomplete. A new more comprehensive interactional model, complementing the extant models, is offered with a view to the more homogeneous conceptualization of exercise addiction. Nevertheless, this dual interactional model has a subjective or idiosyncratic component, that interacts with objective situational elements, for which the nomothetic perspective and scholastic research may not account for. Therefore, the message of this paper is that researchers should clearly distinguish between risk for exercise addiction that may or may not end up in morbidity and actual clinical or psychiatric cases of exercise addiction. The scope of the model presented in here is to draw a line between *risk* and *morbidity.* The exercise addiction literature, apart from a few case studies, deals with estimates of risk that may never turn into actual morbidity.
